# Rate and rhythm control strategies for apraxia of speech in nonfluent primary progressive aphasia

**DOI:** 10.1590/1980-57642018dn12-010012

**Published:** 2018

**Authors:** Bárbara Costa Beber, Monalise Costa Batista Berbert, Ruth Siqueira Grawer, Maria Cristina de Almeida Freitas Cardoso

**Affiliations:** 1 Atlantic Fellow for Equity in Brain Health of the Global Brain Health Institute, Trinity College Dublin, Ireland; 2 Department of Speech and Language Pathology, Federal University of Health Sciences of Porto Alegre, RS, Brazil; 3 Irmandade Santa Casa de Misericórdia de Porto Alegre, RS, Brazil

**Keywords:** speech and language therapy, primary progressive aphasia, rehabilitation, apraxia of speech, fonoaudiologia, afasia progressiva primária, reabilitação, apraxia de fala

## Abstract

The nonfluent/agrammatic variant of primary progressive aphasia is characterized by apraxia of speech and agrammatism. Apraxia of speech limits patients' communication due to slow speaking rate, sound substitutions, articulatory groping, false starts and restarts, segmentation of syllables, and increased difficulty with increasing utterance length. Speech and language therapy is known to benefit individuals with apraxia of speech due to stroke, but little is known about its effects in primary progressive aphasia. This is a case report of a 72-year-old, illiterate housewife, who was diagnosed with nonfluent primary progressive aphasia and received speech and language therapy for apraxia of speech. Rate and rhythm control strategies for apraxia of speech were trained to improve initiation of speech. We discuss the importance of these strategies to alleviate apraxia of speech in this condition and the future perspectives in the area.

## RESUMO

A variante não-fluente/agramática da afasia progressiva primária caracteriza-se por apraxia de fala e agramatismo. A apraxia de fala limita a comunicação devido a redução no fluxo de fala, substituição de sons, tentativas articulatórias, falsos inícios de fala e reinícios, segmentação de sílabas, e aumento da dificuldade conforme o aumento do enunciado. A terapia fonoaudiológica é benéfica em casos de apraxia de fala decorrentes de acidente vascular cerebral, porém pouco se sabe sobre seus efeitos na afasia progressiva primária. Este artigo é um relato de caso de uma mulher de 72 anos, analfabeta e trabalhadora do lar, que foi diagnosticada com afasia progressiva não-fluente e recebeu terapia fonoaudiológica para apraxia de fala. Estratégias de controle do ritmo e da taxa de fala foram utilizadas a fim de melhorar a iniciação da fala. A importância de estratégias para minimizar a apraxia de fala e as perspectivas futuras na área são discutidas neste artigo.

Primary Progressive Aphasia (PPA) is a progressive disorder of language associated with atrophy of frontal and temporal brain regions.^1^^,^ 2 There is no cure for PPA and the treatment is symptomatic. Social interventions, including counseling and speech and language therapy (SLT), help patients and families adapt to the neurocognitive deficits.^3^ The current diagnostic criteria classify PPA into three variants: semantic, logopenic, and nonfluent/agrammatic.^1^ The semantic variant of PPA presents with impairment of word comprehension, object knowledge, and word retrieval, associated with brain atrophy or hypoperfusion of the anterior temporal lobes.^1^ The logopenic variant is characterized by impairment of phonological processing, slow speech rate and pauses secondary to word-retrieval difficulty, and is a consequence of atrophy or hypoperfusion in the left temporoparietal junction.^1^^,^ 4 The last variant is the nonfluent/agrammatic variant of PPA (nfvPPA) and is the focus of this article. Apraxia of speech (AOS) and agrammatism are the core features of nfvPPA due to left posterior frontotemporal region atrophy or hypoperfusion.^1^^,^ 5 AOS is a common symptom in nfvPPA that limits patient communication and significantly compromises quality of life. It is characterized by slow speaking rate, sound substitutions, articulatory groping, false starts and restarts, segmentation of syllables, and increased difficulty with increasing utterance length.^6^^,^ 7

Patients with AOS might benefit from SLT, but we found only one description of a specific AOS treatment (oral reading technique) for nfvPPA in the literature.^8^ However, there are many studies describing the effect of different speech and language therapy approaches for AOS in post-stroke patients.^9^^-^ 12

The American Speech-Language-Hearing Association (ASHA) describes treatment options for acquired AOS. The rate and rhythm control approaches, or prosodic facilitation approaches, are a group of SLT approaches which uses intonation patterns to improve speech production.

Melodic intonation therapy (MIT) and some of its modified versions number among the rate and rhythm control approaches for AOS.^9^^,^ 10 ^,^ 13 MIT combines mainly two components, the melodic intonation of words and single phrases, and rhythmic hand tapping that accompanies the production of each syllable and serves as a catalyst for fluency.^14^ Two hypotheses may help to interpret the effects of MIT in AOS: MIT engages areas for articulation and speech output in the right hemisphere, or MIT engages preserved brain areas in both hemispheres that are usually involved in music processes to contribute to speech processes.^14^ Modified versions of MIT, or techniques that use its principles, are frequently used in the SLT routine.

There is a need for studies that verify the effects of rate and rhythm control approaches in the treatment for AOS in nfvPPA. These approaches might be useful as strategies to alleviate dysfluency. Here, we use a case report to describe strategies that were successfully used to reduce AOS in a patient with nfvPPA.

## CASE REPORT

A 72-year-old, illiterate housewife was referred to an SLT service for speech and language treatment in 2015. She was diagnosed with nfvPPA according to the current diagnosis criteria^1^ in 2012 by the outpatient neurology service of the same hospital.

Cognitive screening was performed with the Mini-Mental State Examination (MMSE)^15^ and with the Clinical Rating Scale (CDR).^16^ She had an MMSE score of 18 and a CDR score of 1. The patient presented normal swallowing when assessed with an adapted version of the Gugging Swallowing Screen (GUSS).^17^ A motor speech assessment was conducted to verify the presence of apraxia, using a qualitative instrument that allows the identification of apraxia signs. In the verbal apraxia assessment, this instrument includes repetition of words, repetition of sentences, emission of automatisms, spontaneous speech and reading aloud. In the nonverbal apraxia assessment, the instrument evaluates orofacial structure movements.^18^ The patient presented with a significant and limiting difficulty to start speech, mainly characterized by blocks and repetitions of sounds. In addition, during the nonverbal tasks, she presented with incoordination of lips, tongue and facial movements.

Language was assessed with an informal spontaneous speech evaluation and the Brazilian Montreal Toulouse Language Assessment (MTL-BR).^19^ During spontaneous speech, features of AOS were again noted, such as false-starts, blocks and repetitions, that prevented her from completing the words and consequently from uttering phrases. In rare situations, when the patient was able to emit sentences, agrammatism was also evident. She was unable to perform writing and reading tasks due to her illiteracy. She had relatively spared oral comprehension and her difficulties occurred when she was presented with grammatically complex sentences. Reduction in speech fluency was evident on the oral narrative task, as well as impairment on the nonverbal praxis task. In the naming task, she was able to recognize the pictures and to show with gestures how to make use of objects, indicating spared semantic abilities. The scores obtained on the language assessment are described in Table 1. This language battery does not provide norms for the illiterate population, and therefore two standard deviations from the lowest level of education was used to verify impairment on the tasks.

**Table 1:** Description of patient's performance on language assessment

Task	Score obtained	Maximum score	Norms for 2 SDs
Structured interview	26	26	24.43
Automatic speech (form)	4	6	4.59
Automatic speech (content)	4	6	6.00
Auditory comprehension (words)	5	5	5.00
Auditory comprehension (sentences)	11	14	8.60
Oral text comprehension	5	9	2.33
Oral narrative (number of words)	7	-	19.79
Oral narrative (time in seconds)	150	-	26.40
Oral narrative (level of difficulty)	3 (severe)	3	-
Repetition (words)	11	11	7.94
Repetition (sentences)	20	22	20.97
Semantic verbal fluency (animals in 1.5 minutes)	12	-	6.07
Nonverbal praxis	18	24	23.42
Naming - nouns	21	24	20.27
Naming - verbs	6	6	3.34
Object manipulation	15	16	15.42
Body part recognition	8	8	8

SD: standard deviation.

The SLT assessment concluded that the impairment in speech production was mainly due to AOS and was the most limiting aspect of her condition. The main feature of AOS in this patient was difficulty initiating speech. Thus, the goal of the speech therapy was to reduce repetitions and blocks to facilitate speech initiation. The patient engaged in 45-minute SLT sessions once per week. She received weekly assignments to be done at home, which were recommended to be performed once a day. The patient's husband was actively involved in the home assignments to guarantee following of the written instructions because the patient was illiterate.

Two techniques of rate and rhythm control approaches were used as strategies to facilitate speech fluency in the initiation of speech. First, the patient was trained to lengthen the first sound or syllable of words when she encountered difficulty initiating emission. Second, the therapist provided a rhythm (by beating hand on the table) and the patient was asked to emit each syllable of the words following the rhythm. It is important to highlight that these techniques have the same rehabilitation principles as the MIT (described in the Introduction section), but do not follow the rules of the original protocol of this therapeutic method. From the fourth session of training, the rhythm was used to mark each word in sentences, so as to increase the complexity of this strategy. The patient was able to use the strategies by herself after two months of training.

Post-intervention assessment was not performed because the patient had a fall that prevented her from continuing to participate in the therapy. Thus, the treatment effects were based on qualitative information, which included observation and reports by the patient and her husband. Thus, we observed that, at the end of the therapy, the patient still presented with AOS, but the strategies allowed her to initiate speech in many situations and to then produce single words and short sentences.

This case report is part of a research project approved by the research ethics committee of the institution (register number 042/12) The participant signed an informed consent form.

## DISCUSSION

Rate and rhythm control approaches for AOS are already known to have positive effects for recovery from nonfluent aphasia in post-stroke patients.^9^^,^ 10 ^,^ 20 ^-^ 22 We believe that these same approaches can be used in the treatment of AOS secondary to neurodegenerative conditions, but with different objectives. The objective of SLT in stroke patients is to recover speech function. In neurodegenerative patients, the objective is to adapt the individual to a new communication condition and employ the same techniques used as exercises in stroke patients as strategies to reduce the impact of the speech disorder on their daily lives. Thus, this case report suggests that speech strategies which follow the principles of the MIT might benefit patients with nfvPPA.

AOS results from damage to brain networks involved in speech planning and programming. The neuronal substrates of AOS remain unclear, where the condition has been attributed to lesions in different cortical areas, including Broca's area, the premotor and supplementary motor areas of the dominant hemisphere, the parietal lobe of somatosensory cortex, the supramarginal gyrus and insula.^23^ The articulatory disorder that occurs in AOS is marked by difficulty in programming the movements for volitional production of phonemes.^23^ This mechanism is impaired in individuals with AOS, leading to blocks or repetitions that hamper the initiation of speech. When a strategy to initiate the speech is presented to the apraxic patient, (i.e. lengthening the first syllable, and following the rhythm of beats to emit the syllables), they have to initiate speech using a different pattern, which needs more conscious and voluntary engagement. During the use of these strategies, the individual needs to follow the pattern of speech provided by the strategy, and this pattern engages different neuronal circuitry, which might be better preserved than the circuitry involved in the initiation of speech under normal conditions of spontaneous speech.

The speech strategies reported in this article were applied in an illiterate woman. This case report highlights the importance of specific strategies for illiterate patients, since techniques predominantly involving reading or writing, such as that reported in the study of Henry et al (2013),^8^ may be useful for most patients but unfeasible for the illiterate population. Furthermore, it calls attention to the importance of family participation in the therapy process, given the need for training the speech strategies at home by following written instructions.

Also regarding illiteracy, one could argue that illiterate patients cannot perform speech techniques that rely on syllable segmentation because they do not hold the knowledge about this linguistic concept. However, we consider that, in order to perform syllabic segmentation of a word, it is necessary to have phonological awareness rather than reading and writing skills. We confirmed this assumption in this case report since the patient was able to perform the tasks.

Our study has relevance in that the literature on language and speech rehabilitation in PPA remains scarce. However, it is an anecdotal study and has some important limitations. First, we could not perform a post-intervention assessment, because the patient had a fall that prevented her from continuing to participate in the speech therapy sessions. Second, dementia severity was investigated using the CDR. However, this instrument might not be suitable for assessing PPA severity because it does not include the language domain, and therefore our data may not accurately represent the severity of this case. Future investigations in Brazilian population should assess severity using the Frontotemporal Dementia Rating Scale (FTD-FRS).^24^

We suggest that future studies investigate the effect of rate and rhythm control strategies to improve speech praxis in groups of patients with AOS secondary to PPA, but also due to other neurodegenerative diseases such as Parkinson's disease, supranuclear palsy, and corticobasal syndrome. Experimental studies comparing pre- and post-intervention conditions may help elucidate the true contribution of these strategies, where randomized clinical trials would be the ideal methodology for such research.
